# Acoustic Sensors for Monitoring and Localizing Partial Discharge Signals in Oil-Immersed Transformers under Array Configuration

**DOI:** 10.3390/s24144704

**Published:** 2024-07-20

**Authors:** Yang Wang, Dong Zhao, Yonggang Jia, Shaocong Wang, Yan Du, Huaqiang Li, Bo Zhang

**Affiliations:** 1 School of Electronics and Information, Xi’an Polytechnic University, Xi’an 710048, China; wangyang@xpu.edu.cn (Y.W.); 220421196@stu.xpu.edu.cn (D.Z.); jiayonggang@stu.xpu.edu.cn (Y.J.); 230421208@stu.xpu.edu.cn (S.W.); duyan@xpu.edu.cn (Y.D.); 2 State Key Laboratory of Electrical Insulation and Power Equipment, Xi’an Jiaotong University, Xi’an 710049, China; lhqxjtu@xjtu.edu.cn

**Keywords:** transformer, multiphysics field simulation, partial discharge, ultrasonic signals, sensor array, location

## Abstract

Partial discharge (PD) is one of the major causes of insulation accidents in oil-immersed transformers, generating a large number of signals that represent the health status of the transformer. In particular, acoustic signals can be detected by sensors to locate the source of the partial discharge. However, the array, type, and quantity of sensors play a crucial role in the research on the localization of partial discharge sources within transformers. Hence, this paper proposes a novel sensor array for the specific localization of PD sources using COMSOL Multiphysics software 6.1 to establish a three-dimensional model of the oil-immersed transformer and the different defect types of two-dimensional models. “Electric-force-acoustic” multiphysics field simulations were conducted to model ultrasonic signals of different types of PD by setting up detection points to collect acoustic signals at different types and temperatures instead of physical sensors. Subsequently, simulated waveforms and acoustic spatial distribution maps were acquired in the software. These simulation results were then combined with the time difference of arrival (TDOA) algorithm to solve a system of equations, ultimately yielding the position of the discharge source. Calculated positions were compared with the actual positions using an error iterative algorithm method, with an average spatial error about 1.3 cm, which falls within an acceptable range for fault diagnosis in transformers, validating the accuracy of the proposed method. Therefore, the presented sensor array and computational localization method offer a reliable theoretical basis for fault diagnosis techniques in transformers.

## 1. Introduction

The power transformer is one of the most critical components in power systems. Hence, failures in transformers can lead to partial power outages and even threaten the overall security of the power grid, causing inconvenience to the public and losses to industries. Recent investigations have highlighted partial discharge (PD) as a major cause of transformer failures, particularly in transformer winding and insulation [1]. Therefore, PD is considered as a significant factor in transformer insulation accidents in both academic and manufacturing fields. With the continuous development of online monitoring and fault diagnosis technology for power transformers, PD has been identified as a key indicator for assessing transformer insulation conditions [2,3].

When PD occurs in transformers, the discharge source releases a large number of signals representing its internal health status. In addition, physical and chemical changes also occur in the vicinity of the discharge including electromagnetic fields, acoustic waves, optical effects, and chemical gases. Although these changes can be detected by specific sensors, the characteristics of these signals are extremely weak and transient. Therefore, due to the fact that partial discharge generates numerous physical reactions and chemical changes, various research fields have proposed a series of methods for detecting partial discharge.

This includes ultrasonic testing (UT) based on acoustic emission signals and acoustic propagation characteristics [4,5], ultra-high frequency (UHF) detection based on UHF signals [6,7], dissolved gas analysis (DGA) for detecting characteristic gases in transformer oil [8], optical detection (OD) using optical probes to detect ultraviolet light [9], and pulse current testing utilizing coupled capacitance, detection impedance [10,11], and ultra-high frequency detection [12,13]. Since various interference signals exist during on-site detection, the obtained signals must be processed in a special way to extract true and effective discharge signals. Moreover, on-site PD detection often requires power outage procedures, consuming significant manpower, materials, and financial resources. Therefore, online monitoring technology is particularly crucial to avoid these drawbacks [14,15].

Markalous et al. [16] conducted research on the physical changes generated by PD, analyzing the process of electromagnetic wave propagation and explaining the propagation principle of the generated pulsed signals by the discharge source. Shuangrui Jia et al. [17] studied the optical effects caused by PD, comparing the optical effects method with the pulsed current method. They concluded that the optical effects method could characterize the magnitude of PD through an integration approach. Moreover, they utilized the phase of the optical signal influenced by insulation defects to decompose the phase resolved partial discharge (PRPD) characteristics and identify the defect types based on the optical signal. The drawback of the pulsed current method is the inability to calculate the discharge source location, while the drawback of the optical effects method is its susceptibility to the influence of deposits in the oil, leading to the reduced sensitivity of OD. Other studies have explored alternative methods to investigate the issue of PD source location such as using high-frequency current transformers, UHF, and specific acoustic sensors [18,19].

Wang and Sorokhaibam [20,21] applied finite element methods (FEMs) to analyze the acoustic waves generated by PD sources in transformers. They arranged a sensor array to select the four sensors with the shortest acoustic wave time and calculated the discharge source coordinates based on the collected acoustic waves. Acoustic wave sensors are evidently less affected by the accumulation of substances in transformer oil but may be susceptible to environmental noise. Markalous et al. [16] provided a scheme for identifying noisy acoustic waves, improving the robustness of acoustic wave sensors during operation. Hekmati et al. [22] calculated the location of the PD source in transformer models using the principles of sound propagation and proposed the precision of PD sources calculated by various sensor arrays. Furthermore, some researchers have integrated optimization algorithms to enhance the accuracy of computing PD source locations [23].

The current methods commonly used for transformer partial discharge monitoring and localization are the UHF and AE methods. The acoustic and UHF localization methods are implemented via piezoelectric sensors and antenna arrays, respectively. However, UHF is more susceptible to electromagnetic interference (EMI) in the field. In addition to distortion, attenuation, and reverberation during signal propagation, field EMI is a major factor contributing to significant localization errors [24]. For ultrasonic sensor localization research, although ultrasonic sensor-based partial discharge localization technology has certain advantages in transformer partial discharge detection, it still faces technical challenges such as signal attenuation, multipath effects, noise interference, sensor placement, and calibration. Through further research and technological advancements, these issues can be gradually overcome, enhancing the effectiveness and reliability of ultrasonic sensor-based partial discharge localization technology.

In this paper, an acoustic sensor array configuration using a six-element cross-shaped microphone array was proposed to address the issue of localizing sources of AE from various types and locations of PD in transformers. By combining the time delay estimation algorithm, the specific coordinates of the PD source can be calculated. This study used COMSOL Multiphysics software to establish models of oil-immersed transformers, sensor arrays, and defect models, simulating four different positions of PD sources along with the corresponding typical PD defects. Subsequently, collecting and analyzing the acoustic signals generated by discharge sources under different types of defects and temperature, and the specific locations of the discharge sources are determined through computation. It should be noted, however, that our proposed method does not take into account the influence of sound transmission through the transformer tank wall, so it is more appropriate for power transformers with built-in sensors [25,26,27]. Despite the fact that they are more expensive and difficult to install, minimizing the computation of transverse waves and reducing the magnitude of errors associated with calculating acoustic wave attenuation could lead to improved localization accuracy.

## 2. Finite Element Analysis of the Generation and Propagation Characteristics of Partial Discharge Ultrasonic Waves

### 2.1. Generation Mechanism of Acoustic Waves in Oil-Immersed Transformers

Through the coupling of “electric-force-acoustic” multiphysics fields and the case analysis of air-gap discharge, it can be observed that a design bubble exists in the winding and insulation paper interlayer. In the absence of local discharge, the relatively stable electric field force compresses the bubble, triggering the elastic force of the bubble itself. Combined with the gravity and friction of the bubble, the environment in which the bubble exists shows a stable state.

**Figure 1:**
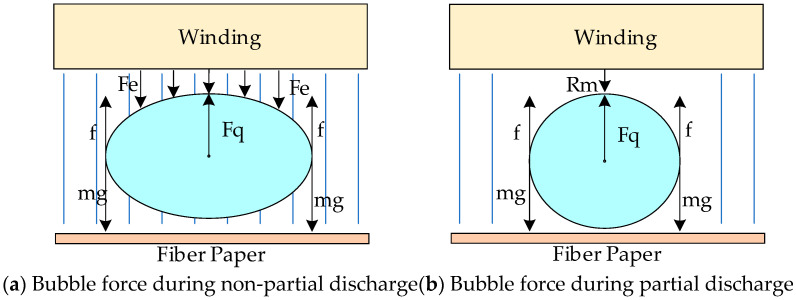
Bubble force analysis diagram.

Through force analysis based on Figure 1a, it can be deduced that the electric field force *F_e_* acting on the bubble counteracts with its own mass m, while the bubble’s elastic force F_q_ and frictional force *f* balance each other, resulting in a stable state. When local discharge occurs, the electric field force originally exerted around the bubble instantaneously disappears, F_q_ = 0, and the discharge time is very short. The bubble cannot undergo an abrupt change to its original state in such a short time. At this point, the equilibrium state of the bubble is suddenly disrupted, leading to high-frequency vibration of the bubble. During the rebound process, the forces acting on the bubble are shown in Figure 1b. The elastic force direction of the bubble is defined as the positive force, while the opposite direction is defined as the negative force. At this time, the bubble is influenced by the elastic force F_q_ as the positive force, and by the frictional force and its own gravitational force as the negative forces.

**Figure 2:**
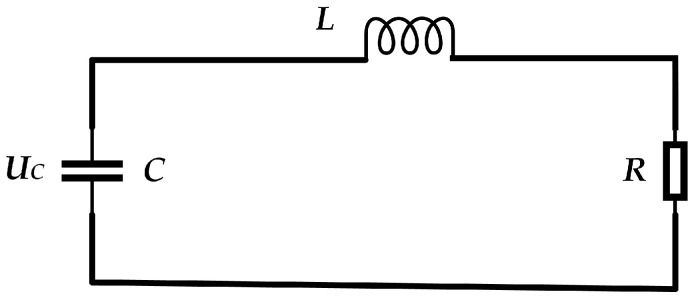
Equivalent circuit diagram.

Using the principle of “electric-force-acoustic” field coupling, an equivalent circuit diagram was constructed, as shown in Figure 2. In this diagram, the resistor *R* corresponds to the mechanical negative force elements acting on the bubble, the inductor *L* corresponds to the mass of the bubble, and the capacitor *C* corresponds to the positive force elements acting on the bubble.

Based on the circuit diagram, the loop equation can be derived according to Kirchhoff’s voltage law:

(1) $$LC\frac{d^{2}U_{c}}{dt^{2}}+RC\frac{dU_{c}}{dt}+U_{c}=0$$

Under normal circumstances, when partial discharge occurs, the gas inside the bubble undergoes vigorous movement, resulting in friction with the bubble wall. However, the frictional force is not significant. In the corresponding circuit, it can be observed that $R<2\sqrt{\left(L/C\right)}$. Therefore, this circuit can be regarded as an oscillation circuit.

The intensity of the acoustic wave is proportional to the energy released by the PD source. Therefore, the amplitude of the acoustic wave is proportional to the square root of the PD energy, indicating a linear relationship between the wave amplitude and the discharge intensity. As the acoustic wave propagates through the insulating medium, its intensity gradually decreases with increasing distance from the PD source. This attenuation is particularly severe after multiple reflections, refractions, and propagation along the steel plate walls of the transformer. Ultrasonic characteristics related to discharge quantity include signal amplitude, signal frequency, and signal duration [28]. With increasing discharge, the ultrasonic spectrum shifts toward lower frequencies. Therefore, from the above analysis, various characteristics of PD acoustic waves can be obtained for further analysis.

### 2.2. Propagation Principle of Sound Wave in Oil Immersed Transformer

When PD occurs inside a transformer, the ultrasonic signals generated propagate through the insulating oil and various core components. The total energy of the sound waves gradually decreases over the unit area as they spread, and the sound wave energy per unit area reduces with the square of the distance from the sound source. In classical acoustical pressure analysis, the momentum equation and continuity equation that govern the propagation of sound waves are as follows:

(2) $$\frac{\partial \vec{v}}{\partial t}+(\vec{v}\cdot \nabla )\vec{v}=-\frac{1}{\rho }\nabla P$$

(3) $$\frac{\partial \rho }{\partial t}+\nabla \cdot (\rho \vec{v})=0$$

where *v* represents the propagation velocity field, *ρ* denotes the medium density, and *P* indicates the medium pressure. In addition to the insulation oil, the internal structure of an oil-immersed transformer is complex. The ultrasonic signals generated by PD sources cannot be directly received by sensors, but instead undergo refraction and absorption by the coils and iron core before reaching the sensor. As a result, the sound wave signals at this point will be accompanied by a certain degree of reverberation, and the waveform will also undergo distortion and attenuation. However, since the amplitude and frequency of the ultrasonic signals generated by PD sources are significantly different from the surrounding interference signals, the surrounding interference variables can be considered small disturbances. This can be represented by Using the principle of “electric-force-acoustic” Equations (4) and (5):

(4) $$S_{i}(t)=p_{i}(t)+n_{i}(t)$$

(5) $$S_{i+1}(t)=\alpha p_{i}(t+\nabla t)+n_{i}(t+\nabla t)$$

where *S_i_* (*i* = 0, 1, …5) represents the mixed acoustic wave signal received by the sensor, *α* denotes the attenuation coefficient of the energy of the sound wave signal received by the sensor, *p_i_* denotes the acoustic energy generated by the PD source, *n_i_* indicates a Gaussian white noise with a mean of 0 and variance of 1, and Δ*t* symbolizes the arrival time difference of the acoustic wave signals received by every two sensors. It is important to note that the white noise *n_i_* and the source sound *p_i_* are independent. By combining the momentum and continuity equations, considering the white noise situation, and then reorganizing and ignoring subtle variations, the wave equation for the acoustic wave can be obtained as follows:

(6) $$\frac{1}{\rho c^{2}}\frac{\partial^{2}P}{\partial t^{2}}-\nabla \cdot \left(-\frac{1}{\rho }\left(\nabla P-q_{d}\right)\right)=Q_{m}$$

where the sound source signal represented by *q_d_* is a dipole domain source, *Q_m_* represents a monopole domain source, and *c* denotes the sound speed of sound wave in oil, which can be represented below.

(7) $$c=\sqrt{\frac{B}{\rho }}$$

where *B* is the volume modulus. After data query [22], the sound speed in the insulation oil was set at 1420 m/s.

### 2.3. Effect of Different Temperatures on Partial Discharge Signal Propagation

The oil-immersed transformer model includes solid components such as iron core, windings, insulation paper, and oil tank, with the remaining parts filled with Kun25# mineral insulating oil. As transformers are affected by temperature variations during different operating times, loads, and conditions, which in turn influence the propagation speed of sound waves, impacting the sound source localization technology mentioned in this paper, it is crucial to study the sound wave characteristics and localization technology under different temperatures. Most solid materials inside the transformer exhibit isotropic properties. Therefore, the calculation formula for the longitudinal wave velocity signal *V_L_* when sound waves travel through solid materials is as follows:

(8) $$V_{L}=\sqrt{\frac{E\left(1-\sigma \right)}{\rho \left(1-2\sigma \right)\left(1+\sigma \right)}}$$

Transverse wave sound velocity *V_T_* is calculated as:

(9) $$V_{T}=\sqrt{\frac{E}{2\left(1+\sigma \right)\rho }}$$

The formula shows that the factors affecting the longitudinal and transverse wave velocities are not significantly correlated with temperature. Therefore, the impact of temperature variation on the speed of sound in solid components inside the transformer can be considered negligible. In addition, the insulating paperboard serves as insulation protection around the windings, with its internal material being cellulose fibers, hence lacking isotropic properties. The direction and velocity of sound wave propagation in cellulose fibers are influenced by fiber orientation, albeit to a small extent. However, the impact of temperature on the paperboard is also minimal. Therefore, the propagation velocity of sound waves in the insulating paperboard can be assumed to be constant.

**Figure 3:**
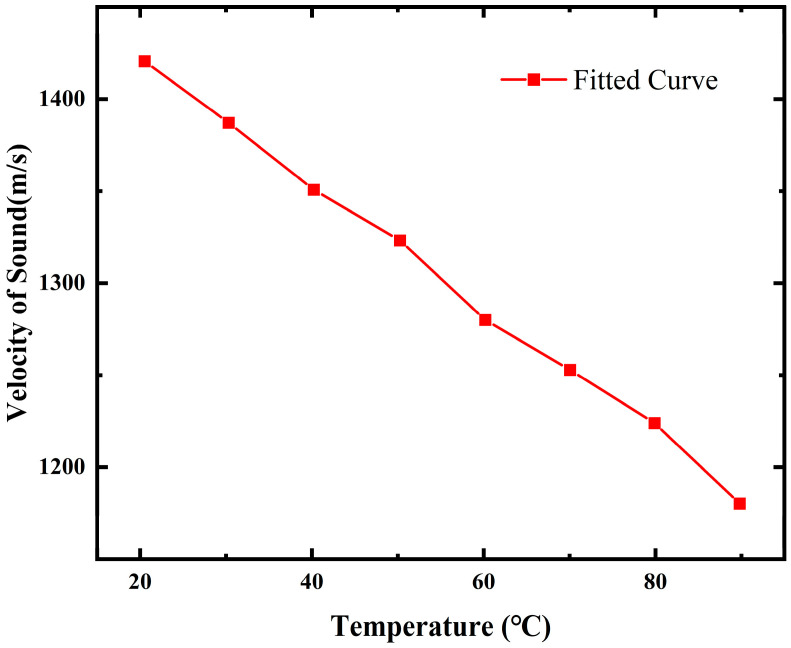
The speed of sound at different temperatures.

Insulating oil, which constitutes the largest proportion of the internal volume of a transformer, the propagation velocity of sound waves in insulating oil is largely dependent on the temperature. This is because temperature variation affects the physical parameters of the insulating oil, subsequently influencing the density, viscosity, and other properties of the liquid. Additionally, factors such as the frequency of sound waves and the water content in the oil also impact the propagation velocity of ultrasonic waves. The main components of insulating mineral oil mainly consist of hydrocarbons, making it a type of Kneser liquid hydrocarbon substance. The propagation velocity of ultrasonic waves decreases with a decrease in the number of molecules. With increasing temperature, the density decreases, reducing the number of molecules, and consequently lowering the propagation velocity of ultrasonic waves. In this paper, simulations of the time of arrival of sound waves at different temperatures were conducted, with the simulated curves shown in Figure 3.

The graph shows that the propagation velocity of sound waves in transformer oil decreased with an increase in temperature, with the curve fully reflecting the relationship between sound velocity and insulating oil temperature. This simulation only considered the effect of temperature on sound velocity under conditions of stationary oil flow, providing sufficient analysis of the impact of different temperatures on partial discharge localization.

## 3. Establishment of Three-Dimensional Models of Transformers and Defective Devices

### 3.1. Finite Element Analysis of Transformer PD Source in Pressure Acoustics

**Figure 4:**
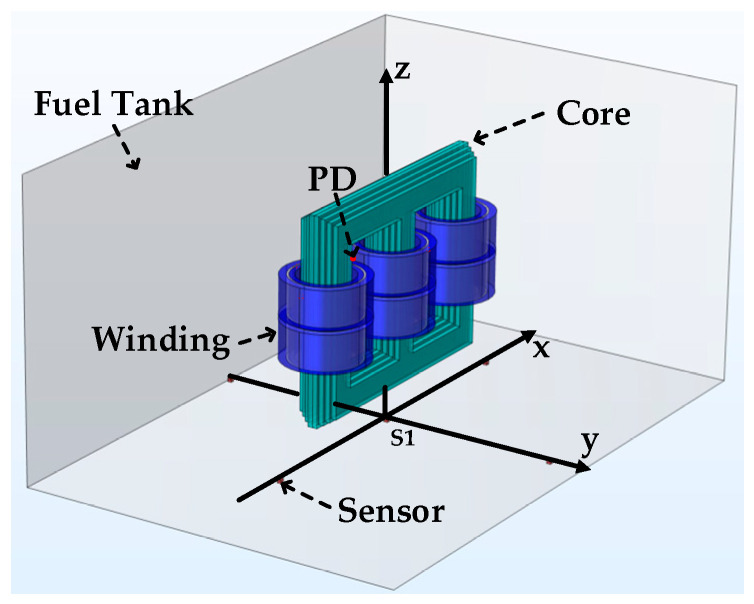
3D transformer model.

This study established a transformer model in three-dimensional space using COMSOL Multiphysics software. The oil tank was constructed using steel plate material filled with insulating oil maintained at a constant temperature of 20 °C. The transformer core and winding materials were specified as silicon steel sheets and copper wires, respectively. All of these materials can be added and set in the software material library. The specific model is shown in Figure 4.

**Figure 5:**
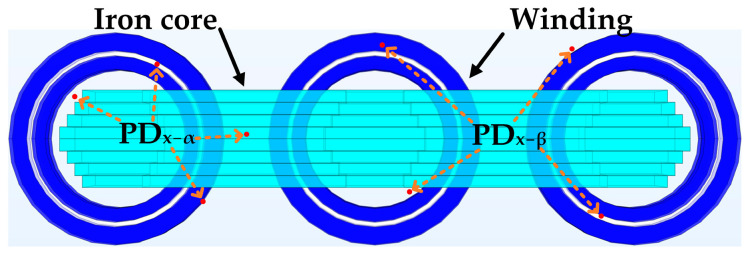
Distribution of partial discharge points in the top view of a transformer.

By analyzing the experimental simulation of the ultrasound signal, COMSOL Multiphysics software can simulate ultrasonic signals during PD using a setting for monopole acoustic wave emission. Therefore, the model was configured with a monopole domain source serving as the acoustic wave emission device, indicating that the sound waves emitted from the PD source are isotropic and time-varying pulse signals. The monopole domain source sphere emits equivalent acoustic radiation in all directions by alternately introducing and removing fluid to generate sound waves in the surrounding area. Within the acoustics domain model, the outer boundary of the transformer oil tank was set as a perfectly matched layer to be equivalent to an impedance form. This setup enables the refractive and absorptive behavior of transmitted acoustic wave signals. The position of the PD source in the model is shown in the top view in Figure 5.

Figure 4 and Figure 5 depict the three-dimensional model of the transformer in space and a top view of the internal core components of the transformer, respectively. In actual transformers, insulation paper and other components are between the windings. In the field of acoustics, insulation paper acts as an obstacle on the acoustic wave transmission path, absorbing a small fraction of sound waves, leading to a decrease in the amplitude of the sound waves received by the sensor. However, it does not distort the sound wave signal, ensuring that the sound wave signal maintains a reliable signal-to-noise ratio after passing through the insulation paper. The specific parameters utilized for the finite element model are shown in Table 1 and Table 2.

**Figure 6:**
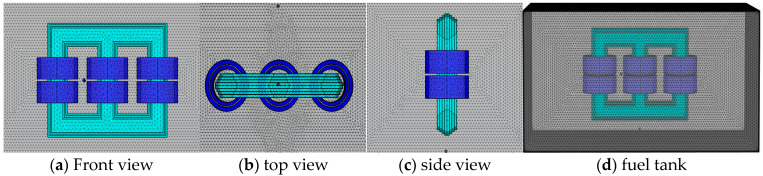
Grid diagram.

As this paper mainly focused on the internal acoustics propagation within the transformer, all material settings were assumed to be isentropic idealizations without considering their losses and heat generation. After setting the model and material parameters, the internal core components and PD sources of the transformer were refined using tetrahedral meshes when partitioning the model grid. This refinement aimed to enhance the computational accuracy of the propagation medium from the PD source to the sensor, thus facilitating the attainment of accurate solutions. In contrast, components that were far from the PD source and the sensor such as the transformer tank were assigned relatively sparse grid densities. This decision was based on the understanding that when the distance from the PD source is large, the computational accuracy requirements for ultrasonic signals diminish, thus not necessitating the same level of precision as close to the PD source. This approach also reduces the computational load and saves computation time. The specific grid partition is illustrated in Figure 6.

The total number of grid cells in the mesh was 859,517, with 144,879 grid vertices. The average cell quality was 0.6639, meeting the requirements for grid partitioning.

The wave equation for acoustic waves is expressed by Equation (6). In the model, the acoustic field of the discharge source is treated as a monopole domain source, requiring uniform propagation in all directions within the space, in which the resulting acoustic effect is similar to that of the PD source. The acoustic wave signal emitted from the monopole domain source resembles a damped sine wave characterized by the main frequency range of 20 to 500 kHz [18]. The waveform equation corresponding to the monopole domain source set in the model is shown in Equation (10).

(10) $$Q_{m}(t)=A\left(\exp(-\frac{t}{\tau })\right)\sin(2\pi \cdot f\cdot t)$$

where *Q_m_* represents the monopole source varying with time, and the coefficient *A* is the acoustic energy flow coefficient (assumed to be a constant 1 in this context) representing the attenuation coefficient of the change in acoustic wave energy over time and distance. In this study, the attenuation time was set at 70 ms and decayed to almost zero by 300 ms.

### 3.2. Defective Device Model and Finite Element Analysis

**Figure 7:**
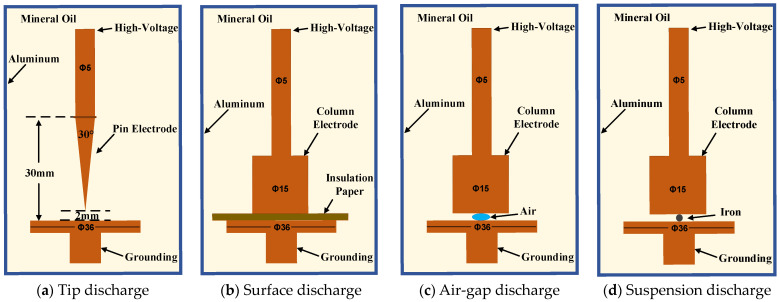
Schematic diagram of simulation model under different partial discharge types.

In this study, utilizing the multiphysics coupling simulation platform of the “electric-force-acoustic” module in COMSOL software 6.1, four typical models of partial discharge simulations were designed based on the common characteristics of insulation defects and discharges in transformers including tip discharge, surface discharge, air-gap discharge, and suspended discharge, as shown in Figure 7.

Connecting the needle electrode and cylindrical electrode to the high-voltage section, grounding was applied to the ground electrode, resulting in a significant potential difference between the electrodes. In the tip discharge model, due to the gradual thinning of the needle plate electrode shape, uneven discharge is generated during the partial discharge process, effectively simulating the tip discharge effect. The cylindrical electrode shape is more regular, with a uniform internal electric field distribution. Surface discharge along the insulation board, air bubble discharge, and suspended discharge were simulated by adding insulation paper, air bubbles, and metal particles, respectively. All insulation paper, electrodes, and metal particles in the model had smooth surfaces, without considering burrs or rough workmanship issues, with the specific dimensions as shown in Figure 7.

Currently, some studies have acquired and analyzed ultrasonic waveforms from four different types of defects using an experimental platform. It was found that the ultrasonic waves generated under four types of defect conditions varied in frequency, amplitude, duration, and waveform smoothness due to the different discharge currents or the heterogeneity of the discharge medium.

**Figure 8:**
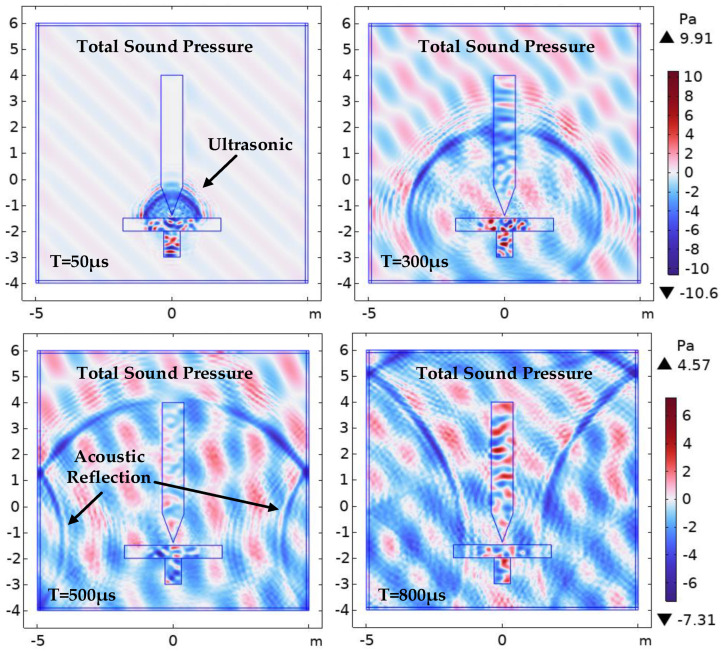
Plane distribution of sound waves under the coupling of “electric-force-acoustic”.

This study primarily focused on localizing partial discharge points inside transformers. Therefore, the four types of defect models established were visualized using a multi-physics simulation approach to analyze the process of solid vibration and subsequent sound wave generation caused by the discharge phenomena. Background pressure fields were added to simulate the actual background interference noise in transformers as the basis of the acoustic environment, efficiently reproducing the sound wave impacts generated by actual partial discharges. From the simulation results, it is evident that, for the purposes of localizing discharge points in this study, the main factor affecting the arrival time of sound waves is the first waveform of the ultrasonic waves. The different waveform characteristics generated under different defect types do not affect the calculation of arrival time. Therefore, at equal signal amplitudes, the type of discharge defect does not affect the effectiveness of sound wave localization techniques. Additionally, sound wave characteristics generated by needle-plate electrodes are more pronounced and easier to achieve. Therefore, this study used needle-plate electrodes as an example to demonstrate the analysis of simulation results, as shown in Figure 8.

When sound waves are generated under the coupling of “electric-force-acoustic” conditions, the waveform initially spreads uniformly in all directions. As the sound waves propagate through the transformer oil and collide with solid electrodes, a small amount of sound waves is absorbed, resulting in a slight decrease in the amplitude trend of the sound waves. However, the main trend of the sound wave waveform and the arrival time of the sound wave will not be affected. According to Equation (10), it can be understood that partial discharge acoustic waves can be treated as oscillatory sound waves, which subsequently yields the formula for solving acoustic waves:

(11) $$U_{c}=\frac{U_{0}\omega_{0}}{\omega }e^{-\delta t}\sin(\omega t+\beta )$$

From the above equation, it can be inferred that the ultrasonic signal of partial discharge is a periodic waveform that undergoes continuous oscillation and attenuation. The continuous vibrations of the bubbles lead to the generation of ultrasonic waves.

## 4. Finite Element Simulation of Local Ultrasonic Propagation Characteristics

Aging insulation and the rough workmanship of transformers are important contributors to PD. Various defects lead to different types of discharge, and the four most common types of PDs are corona discharge, surface discharge, tip discharge, and suspended discharge. The characteristic acoustic signal generated by PD depends on the type of discharge, discharge location, and discharge energy, so performing finite element analysis based on the waveform of sound waves emitted by different discharge types is highly crucial.

### 4.1. Analysis of Sound Wave Form Emitted by PD Source

**Figure 9:**
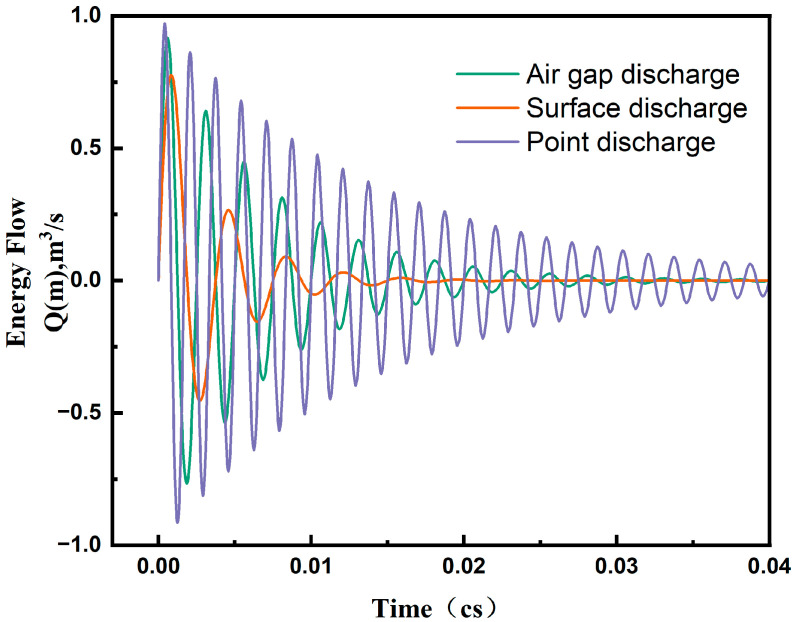
The waveform characteristics of acoustic sources under different types of partial discharge conditions.

These differences in the waveforms of the acoustic signals corresponding to different PD types are reflected in terms of frequency, amplitude, and smoothness. The specific characteristic waveforms of air-gap discharge, surface discharge, and tip discharge were drawn using the editing function module in COMSOL Multiphysics software combined with Equation (11), as shown in Figure 9.

Figure 9 illustrates the characteristic curves of acoustic waveforms over time for different PD types. As can be seen, the surface discharge waveform was smooth with lower frequency and amplitude, typically occurring on the surface of insulation materials. Conversely, the tip discharge waveform was sharp with higher frequency and amplitude, often occurring at sharp electrodes. Corona discharge exhibited an amplitude and frequency between the two, typically occurring in the presence of bubbles. Combined with the analysis of Equation (10), it is known that the acoustic wave generated by the PD source can be equivalent to a monopole source sine wave signal. The monopole source acoustic wave propagated uniformly from the center of the sphere to all faces in three-dimensional space. Analyzing all waveform curves, a maximum peak occurred shortly after the onset of PD, propagating as a sine waveform and decaying over time. The waveform experienced rapid decay in the early stage, with the oscillation amplitude of the waveform approaching zero at approximately 300 ms.

The waveform characteristics of floating discharge acoustic waves are slightly different from other types because they typically occur within insulating materials or media, and various situations require a separate equation or a more complex simulation model. According to data queries, the floating discharge pressure can be calculated by Equation (12).

(12) P(t)=A×exp(−t/sin(2×f×t))

where *p* represents the amplitude of the temporal variation of the acoustic pressure field, *A* indicates the amplitude constant, and *f* denotes the frequency of the acoustic wave.

The PD quantities of our simulated acoustic signals in relation to three PD types can be approximately estimated using the amplitude values of the acoustic waveforms [29], which are within the range of 700–1200 pC. Acoustic signals are commonly believed to be detectable when PD levels are larger than several hundred pC. In other words, the PD represented by our generated acoustic signals can be detected. It is also possible to examine a more quantitative correlation between PD energies and their acoustic signals as indicated in the literature [30] through experiments.

### 4.2. Acoustic Wave Analysis of Sensor Monitoring Points

**Figure 10:**
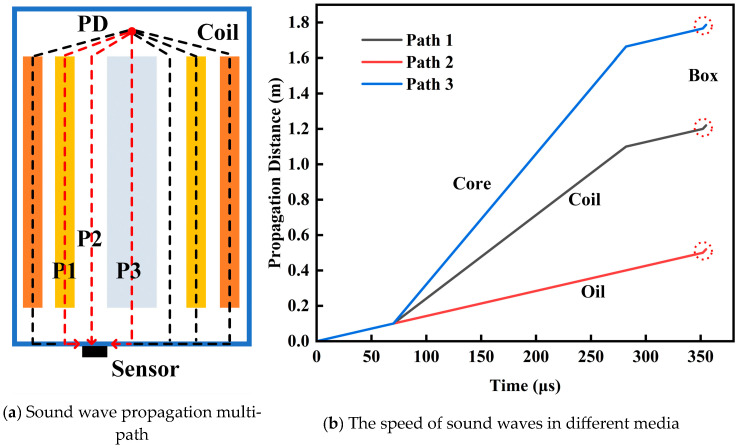
Propagation characteristics of sound waves in different media.

Due to the complex structure inside the transformer, partial discharge ultrasound typically propagates through internal solid components such as the core and windings. The speed of sound in the windings and core is generally much higher than in the insulating oil. When the sound wave reaches the surface of the solid components, reflection and refraction occur. Therefore, the first wave detected by the ultrasonic sensor has often traversed a significant amount of solid medium. As illustrated in Figure 10, the ultrasonic sensor mounted on the outer side of the tank wall receives sound waves corresponding to the wave propagating through the winding medium, the direct wave, and the wave propagating through the core medium, with the main propagation paths corresponding to P1, P2, and P3, respectively.

Due to the speed of sound being greater in the core than in the windings, and greater in the windings than in the insulating oil, it can be observed from the figure that the sequence in which the sensor receives the sound waves is P3, P1, and P2. Therefore, the final waveform received is the superposition of waves from each propagation path, resulting in overlapping waves. Figure 10b describes the relationship between the propagation distances and time of the three paths. The slope of the curve indicates the speed of sound. From the figure, it can be seen that when the sound waves propagate through the core and windings, the slope of the curve is steeper, indicating higher sound speeds. Conversely, sound waves propagating only through the insulating oil have a lower and constant speed. Finally, the sound waves are transmitted to the sensor through the transformer tank. The slope of the sound wave curve within the transformer tank is the steepest, indicating the highest speed, with the propagation path through the tank material usually being the shortest. The propagation speed of sound waves in solid media is high, but the attenuation is also significant.

**Figure 11:**
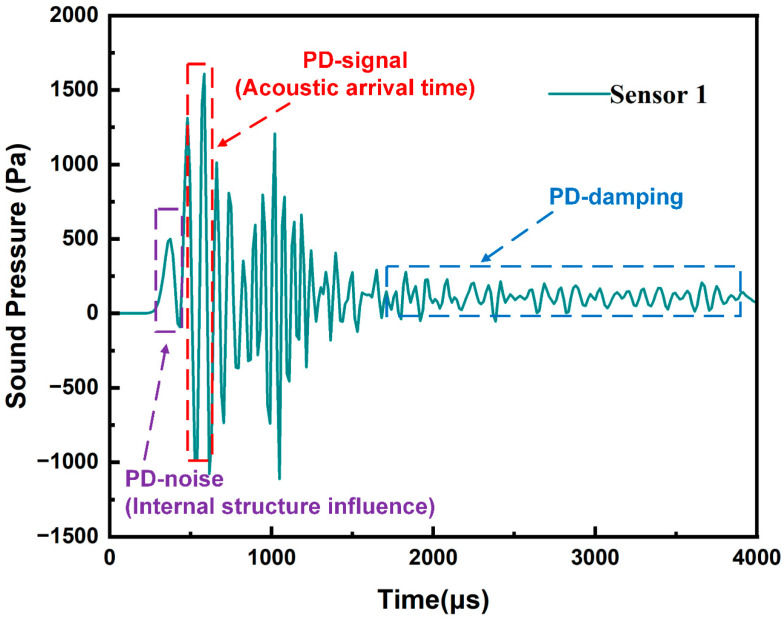
Partial discharge signal simulating the acoustic characteristic signal.

Consequently, the amplitude of the sound waves received by the sensor will be considerably reduced, as evidenced by the decrease in sound wave amplitude observed in the leading edge of the wave in Figure 11.

Figure 11 depicts the acoustic waveform received at the sensor detection point. The initial part of the waveform displays a smooth curve, representing the quiet state within the transformer tank before PD occurs. During this quiet period, simulated interference signals tend toward zero, while actual white noise inside the transformer is stable and with low amplitude similar to the attenuated portion at the end of Figure 11, which did not affect the analysis and calculation in this study. Since the internal structure of the transformer refracts the acoustic wave and interacts with the subsequent new acoustic wave, the first wave peak when the acoustic wave reaches the detection point is often not a pure PD signal, leading to a lower initial peak value of the acoustic wave signal reaching the detection point. Subsequently, there is a peak signal with a significant difference from the previous acoustic wave, and the time point corresponding to this peak is considered as the arrival time of the acoustic wave.

This study initially employed an array of five-element cross-shaped microphones to monitor the acoustic waves from four different discharge source locations. Subsequently, a novel array configuration was utilized for detection and localization purposes. Given the presence of interfering acoustic waves in the detected waveforms, a comparative analysis of the simulated finite element-generated acoustic signals was conducted to identify the characteristic information of the interference signals, facilitating the recognition of interfering acoustic waves in the waveform of the acoustic waves, as theoretically explained in Equation (6). Specific coordinates for each PD source position were set in the COMSOL Multiphysics software, as shown in Table 3.

Table 3 shows four common discharge types, with two common discharge locations set for each type. The coordinates of discharge points in the α rows were set to simulate the detection and localization of different types of local discharge points, while the β rows simulated the impact of local discharge localization under different temperatures. The simulation of local discharge sources in different positions was aimed at enriching the propagation paths of sound waves as much as possible. Taking the case of air-gap discharge in the α row discharge point of PD_1_ as an example, the waveform of the sound waves received at detection point S_1_ is shown in Figure 11.

## 5. Simulation Results and Positioning Calculation Under Six-Element Cross Array

Finite element analysis enables the visualization of the transient spatial distribution and waveform of sound waves, thereby facilitating better data analysis and providing improved parameter references for research on transformer fault diagnosis and other related studies.

### 5.1. Spatial Distribution of Sound Waves

**Figure 12:**
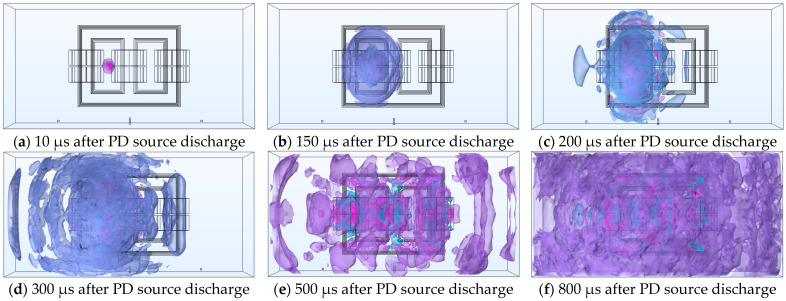
The diffusion distribution of sound waves in three-dimensional space.

Figure 12 shows the spatial propagation iso-surface of the ultrasonic wave signal over time when the PD source S_1_ emits a sound wave signal in an oil-immersed transformer. As seen in Figure 12a, the ultrasonic signal mainly spreads in the form of spherical waves in the surrounding insulation oil, with the highest sound pressure values near the sound source, represented by the red regions in this figure. Upon reaching the winding or iron core, the sound wave signal first undergoes refraction and absorption. The refracted signal and the new signal overlap, resulting in higher sound pressure values. The parts that cancel each other out are shown in lighter colors in this figure. Consequently, there are some red areas and lighter color areas in the simulation results of the transformer core and winding, indicating that the acoustic pressure value is not completely uniform.

Figure 12 shows that after a period of time following PD, the acoustic wave signal rapidly propagated in the insulating oil, and approximately 800 µs after the acoustic wave signal, it nearly filled the entire inner wall of the oil tank. The figure displays the spatial acoustic equal value distribution at time intervals of 100 µs, 150 µs, 200 µs, 300 µs, 500 µs, and 800 µs after the emission of the acoustic wave from the PD discharge source. The diffusion behavior of the acoustic wave in space can be observed from the color difference, gradually attenuating with the distance from the PD source. The amplitude of the acoustic wave also gradually reduced, verifying the diffusion and attenuation principle of the sound source or spherical wave. Under the condition of the constant total energy of the sound source, the energy of the acoustic wave per unit area is inversely proportional to the square of the distance from the sound source, which can be calculated as follows:

(13) $$E_{0}=4\pi \cdot E_{r}\cdot r^{2}$$

where *E*_0_ represents the point sound source intensity, *E_r_* denotes the energy per unit area at a distance r from the sound source, and *r* indicates the distance from the sound source.

During the propagation of sound waves inside a transformer toward the detection point, the waves pass through the iron core and winding sections in most cases. After removing the refraction and reflection of sound waves, the theoretical propagation speed of the waves through the iron core and windings is expected to be faster than in the oil. Therefore, when reaching the detection point, the leading part of the waveform will be accompanied by a certain amount of reverberation. A preliminary explanation is provided in Figure 11.

### 5.2. PD Source Location Calculation

The five-element cross-coordinate system designates the center of the bottom surface of the transformer oil tank as the origin. The coordinates for the positions of the six sound wave sensors are as follows: S_0_ (0, 0, 0), S_1_ (−d, 0, 0), S_2_ (0, d, 0), S_3_ (d, 0, 0), S_4_ (0, −d, 0), and S_5_ (0, 0, h), where *d* represents an appropriate distance ensuring the equidistance of sensors S_1_ to S_4_ from the source point, and *h* represents the symmetric point with respect to the midpoint of the transformer’s height.

**Figure 13:**
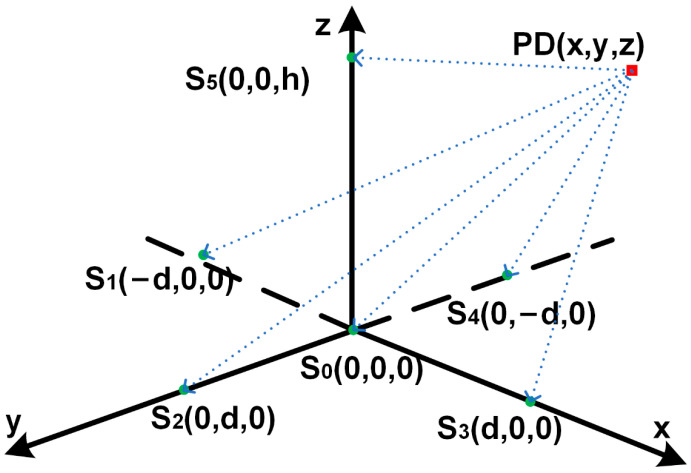
Six-element cross microphone array and power supply diagram.

The specific distribution is illustrated in Figure 13, depicting the positional relationships between the sensors and the PD sources. These four PD sources were located within the low-voltage winding, high-voltage winding, and yoke. Figure 13 provides a schematic overview of the positional relationship between the sensors and the PD sources. Finally, the coordinates of the discharge sources were calculated, and the accuracy of the simulation was validated using error calculation methods.

**Figure 14:**
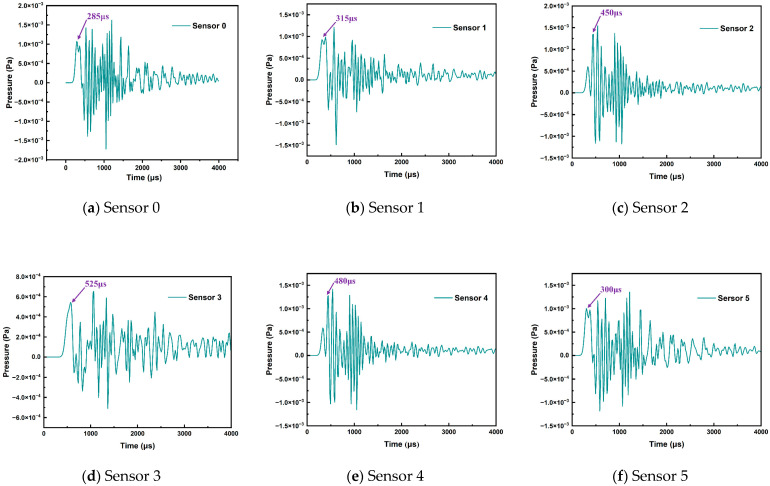
The received S_1_ acoustic wave pattern under the sensor array.

Based on the array of six-element cross sensors, Figure 14 shows the detected sound waveforms at each detection point when the PD discharge source at position S_1_ emits different types of discharges.

Figure 14 shows that the time at which the sound wave first reaches each sensor is different. Therefore, this paper leveraged a direct solution method based on time differences to calculate the location of the PD source. For each plane in three-dimensional space, obtaining n sound wave equations through measurements yields *(n* − 1) time delay equations. Therefore, a minimum of four detection points is necessary for three-dimensional space to obtain 3-time delay equations and determine the three-dimensional spatial coordinates of the PD source. Although the method of locating the sound source with four detection points can be used as a tetrahedral array, the accuracy of the calculation is much lower than that of the five-element cross array. The equation for sound source localization is shown in Equation (14).

(14) $$\left\{\begin{array}{l} x_{pd}^{2}+y_{pd}^{2}+z_{pd}^{2}=R_{0}^{2}\\ {\left(x_{pd}+d\right)}^{2}+y_{pd}^{2}+z_{pd}^{2}=R_{1}^{2}\\ x_{pd}^{2}+{\left(y_{pd}-d\right)}^{2}+z_{pd}^{2}=R_{2}^{2}\\ {\left(x_{pd}-d\right)}^{2}+y_{pd}^{2}+z_{pd}^{2}=R_{3}^{2}\\ x_{pd}^{2}+{\left(y_{pd}+d\right)}^{2}+z_{pd}^{2}=R_{4}^{2}\\ x_{pd}^{2}+y_{pd}^{2}+{\left(z_{pd}-2d\right)}^{2}=R_{5}^{2} \end{array}\right.$$

where *x_pd_*, *y_pd_*, and *z_pd_* represent the coordinates of the PD source, and *d* denotes the appropriate size of the transformer. Here, the length of d was set to 0.6 m, and *d* = 0.5 h. *R_i_* indicates the distance between the PD source and the corresponding sensor. Using sound wave propagation Equation (6), we can obtain:

(15) $$R_{i}=c_{o}\cdot T_{i}$$

where *c_o_* represents the speed of sound in the transformer insulating oil.

In the positional location calculation of the discharge source PD_1_, the coordinates of the PD source can be calculated using the formula by selecting four detection points: S_0_, S_1_, S_2_, and S_5_. Among these, sensor S_0_, with the shortest arrival time, served as the reference detection point for position calculation. Utilizing signals with minimal distortion allows for more accurate calculation results. Furthermore, selecting detection points S_1_ and S_2_, which exhibited significantly larger arrival time differences from S_0_, enhanced the distinctiveness of the time differences in the calculation equations, thereby improving the calculation accuracy.

The signal-to-noise ratio of the detected acoustic wave signals at the detection points is a crucial factor in acoustic analysis. The signal-to-noise ratio represents the ratio of the pure acoustic wave signal emitted by the sound source to other white noise. In normal operation, three-phase AC transformers exhibit a large number of different sizes and shapes of magnetic domains inside the core that constantly change with the AC voltage, resulting in the generation of pulse acoustic waves within the core, known as magnetostriction noise. This type of noise can reach up to several kHz in some cases, thereby affecting the measurement data of acoustic waves. Magnetostriction noise can be eliminated and attenuated using bandpass filters to ensure more accurate measurement results. The positions of different types of PD sources can be calculated using the selected detection points and corresponding calculation methods, as shown in Table 4.

Table 4 displays the calculated coordinates corresponding to different discharge types. Since the discharge source PD_1_ can only occur as corona discharge, Table 4 only includes the calculated coordinates for corona discharge positions in this paper. During the operation of a transformer, losses such as those in the iron core and windings generate a significant amount of heat, leading to an increase in the internal temperature of the transformer. As a result, the physical properties of the insulating oil may change, affecting the speed of sound propagation. In oil-immersed transformers, the temperature of the upper layer of oil typically exceeds 313.15 K during normal operation. For higher loads, the oil temperature can continue to rise, reaching temperatures of over 373.15 K. According to the simulation results in Figure 3, the speed of sound in oil at a temperature of 313.15 K is approximately 1350 m/s. Compared to the speed at room temperature, the percentage difference in speed change ranged from 5% to 17%. Such significant variations in sound velocity can introduce errors in ultrasonic localization technology.

Therefore, in this study, various material parameter characteristics were adjusted at different temperatures to simulate partial discharges of the same type, using tip discharge faults as an example, and conducted localization based on these adjustments. The specific localization coordinates are presented in Table 5.

### 5.3. Error Calculation

**Figure 15:**
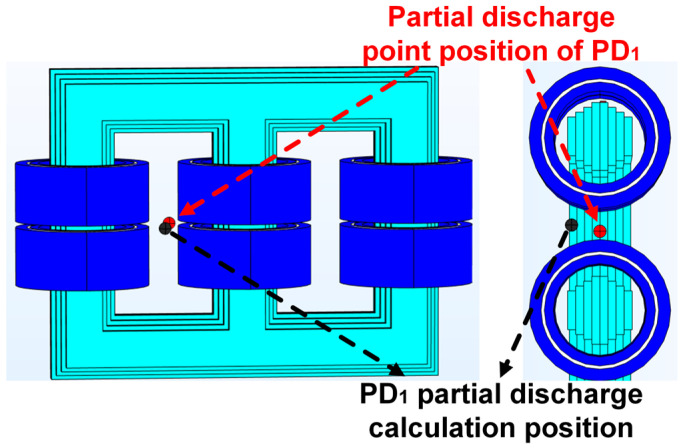
PD_1_ power supply calculation and actual position.

The coordinates of each discharge source were calculated using the method of time difference of arrival by a new type of sensor array. The positioning of these sources on the transformer diagram is shown in Figure 15, where the difference between the calculated positions and the actual positions can be visually observed. Pre-processing of fault diagnosis was performed around the calculated discharge source positions to reduce the occurrence rate of partial discharge, and the sensors in the five-element cross array were located in the same plane, affecting the accuracy of the z-axis coordinate when locating the PD source.

In this study, the sensors were positioned symmetrically with respect to the origin to provide a reference for the z-axis. As a result, the error in the z-component of the calculated results was minimal. This approach enhances the accuracy of determining the coordinates of each PD source.

Figure 15 illustrates the localization results of PD. In this figure, the red marks indicate the actual set positions of the discharge sources, while the black marks represent the calculated positions under the new sensor array. The following equation can be used to compute the distance between the two PD sources.

(16) $$\Delta R=\sqrt{{\left(x^{\prime }-x\right)}^{2}+{\left(y^{\prime }-y\right)}^{2}+{\left(z^{\prime }-z\right)}^{2}}$$

where Δ*R* represents the straight-line distance between the calculated and actual centers of the discharge sources, and *x*′ and *x* denote the calculated and actual abscissae of the discharge sources, respectively. The errors of each PD source, obtained through calculation, are shown in Table 6 and Table 7.

From the statistical analysis of the table, the average error in the overall measurement data between the two centroids was approximately 5 cm. In the simulated measurements, the maximum average error in the calculation of air-gap discharge was around 7.27 cm, while the minimum average error was 2.78 cm. In the simulation of air-gap discharge, the shape of the PD source model was similar to a spherical shape. After removing the 1 cm radius of the actual and simulated discharge spheres, the boundary distance between the two discharge sources was approximately 0.78 cm. As mentioned in Table 1, in a transformer oil tank with internal dimensions of 3 m × 2 m × 1.5 m, the error accounted for 0.5% of the total transformer volume, which sufficiently reflects the accuracy of the new array positioning.

From the calculation table data on errors in positioning research under different temperatures, it can be seen that in this simulation, the highest calculation accuracy was achieved for 293.15 K and 363.15 K. The average error between the calculated coordinates and the actual coordinates was around 4–5 cm. In contrast, the calculation errors for temperatures of 313.15 K and 343.15 K were relatively larger, at approximately 5–6 cm.

### 5.4. Local Partial Discharge Source Positioning Method Based on Dynamic Distance Difference Correction, and Error Iteration Algorithm

Due to the fact that the liquid materials added in the simulation model were all in the ideal transformer oil state, the coordinates of PD calculated by the method of time difference were assumed to be uniformly propagated along a straight-line path by acoustic waves. In reality, however, acoustic waves propagate nonlinearly and non-uniformly. The distances d′_i1_ between the calculated PD source and the sensors were referenced on the assumption of linear acoustic wave propagation. In practice, there exists a certain error between the actual ultrasonic propagation path d_i1_ and d′_i1_, resulting in significant errors in local partial discharge source localization. Therefore, it is necessary to correct d′_i1_ to make it as close as possible to the distance of the actual propagation path.

Let the actual coordinates of the PD discharge source be (*x*, *y*, *z*), and the computed coordinates be (*x*′, *y*′, *z*′). When the acoustic impedance distribution is uniform, the difference between the straight-line distance from sensor i to the PD and the straight-line distance from sensor 1 to the PD is:

(17) $$\begin{array}{l} d_{i-1}=\sqrt{{\left(x-x_{i}\right)}^{2}+{\left(y-y_{i}\right)}^{2}+{\left(z-z_{i}\right)}^{2}}\\ -\sqrt{{\left(x-x_{1}\right)}^{2}+{\left(y-y_{1}\right)}^{2}+{\left(z-z_{1}\right)}^{2}}=d_{i}-d_{1} \end{array}$$

where *d_i_* is the linear distance between the *i*-th sensor and the local power supply.

Therefore, the distance difference obtained by the simulation calculation is:

(18) $$d_{i1}^{\prime }=t_{i1}^{\prime }\times v=d_{i}^{\prime }-d_{1}^{\prime }$$

where *v* represents the constant speed of sound in the oil, and *t*′*_i1_* is the time difference of the acoustic waves between sensor i and sensor 1. Based on the previous calculation, the straight-line distance from the *i*-th sensor to the coordinates of the local discharge source is:

(19) $$d_{str,i}=\sqrt{{\left(x_{ini}-x_{i}\right)}^{2}+{\left(y_{ini}-y_{i}\right)}^{2}+{\left(z_{ini}-z_{i}\right)}^{2}}$$

where *str* represents the physical parameter in the case of linear propagation.

For the actual propagation path of the acoustic waves, it will curve based on the internal structure of the transformer. The main propagation path is divided into N segments, with each segment assumed to have uniform acoustic impedance and no significant angle abrupt changes. Therefore, the distance of the main route can be expressed as:

(20) $$d_{main,i}=v\times t_{main,i}$$

where *main* represents the physical parameters of the main propagation path, and *t_main_* is the propagation time between the local discharge source and the sensors along the main propagation path, expressed as:

(21) $$t_{main,i}=\sum_{n=1}^{N}\left(D_{main,i,n}^{\left[x_{ini},y_{ini},z_{ini}\right]}/v_{i,n}\right)$$

where *D* represents the distance from the localization calculation of the PD location to the *i*-th sensor in the main path of segment n, and *v_i,n_* represents the propagation path of ultrasound in segment n. The error between the main acoustic propagation path distance and the straight-line distance is:

(22) $$\Delta d_{i}=d_{main,i}-d_{str,i}$$

In order to calculate the PD source location more accurately, the value of *d_str,i_* should be brought as close as possible to the value of *d_main,i_*. Therefore, by using the method of analogizing distances with the same properties, these two physical quantities were used to determine the approximate error. Subsequently, the corrected error distance *d*′*_str,i_* can be obtained by subtracting the main path and error Δ*d_i_*.

(23) $$d_{i}^{\prime \prime }=d_{i}^{\prime }-\Delta d_{i}$$

Substitute this into the formula to obtain:

(24) $$\begin{array}{l} d_{i1}^{\prime \prime }=d_{i}^{\prime \prime }-d_{1}^{\prime \prime }=(d_{i}^{\prime }-\Delta d_{i})-(d_{1}^{\prime }-\Delta d)\\ =(d_{i}^{\prime }-d_{1}^{\prime })-\Delta d_{i}+\Delta d_{1} \end{array}$$

where *d*′*_i_* represents the initial calculated distance. Therefore, the above equation can also be simplified as:

(25) $$d_{i1}^{\prime \prime }=d_{i1}^{\prime }-\Delta d_{i}+\Delta d_{1}$$

According to the corrected distance difference *d*′*_i_*_1_, a new set of local power source coordinates with less error can be obtained. The initial coordinates of the local power supply are iterated, and the number of iterations is k.

(26) $$\left[x(k),y(k),z(k)]=f(d_{21}^{\prime }(k),d_{31}^{\prime }(k),d_{41}^{\prime }(k)\right)$$

where *f* () represents the nonlinear functional relationship of the time difference equations. Therefore, the *k*-th iteration of sound wave propagation time in the main line can also be expressed as:

(27) $$t_{main,i}(k)=\sum_{n=1}^{N(k)}\left(D_{main,i,n}^{\left[x(k-1),y(k-1),z(k-1)\right]}(k)/v_{i,n}(k)\right)$$

The corrected distance can therefore be expressed as:

(28) $$d_{i1}^{\prime }(k)=d_{i1}^{\prime }(0)-\Delta d_{i}(k)+\Delta d_{1}(k)$$

Similarly, a new set of local discharge source coordinates can be obtained for *d*′*_i_*_1_(*k*).

(29) $$\left[x(k),y(k),z(k)]=f(d_{21}^{\prime }(k),d_{31}^{\prime }(k),d_{41}^{\prime }(k)\right)$$

Based on the results of multiple calculations, it can be concluded that when the number of iterations reaches around 10 times, the results of the PD source localization tend to stabilize within a small range of oscillation. Therefore, to improve the accuracy and efficiency of the calculations, this study set the total number of iterations to be around 30 to 40 times. The final output result was obtained by averaging the results of the last 10 iterations, and the final expression is as follows:

(30) $$[x_{final},y_{final},z_{final}]=(\sum_{n=k-9}^{k}\left[x(n),y(n),z(n)]\right)/10$$

**Figure 16:**
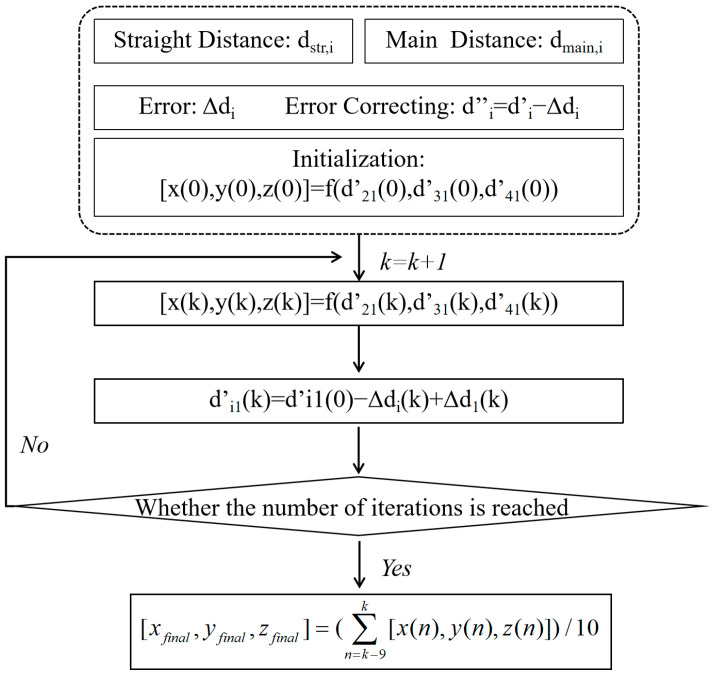
Overall flow diagram.

The overall process flowchart is shown in Figure 16.

Through iterative calculations at different numbers of iterations, it was found that the error was reduced by 26.49% after the first iteration, and decreased to only 25.17% of the original error after the 40th iteration. A significant reduction in calculation error was observed in the first iteration, and as the number of iterations increased, the error was reduced to a quarter of the original value. The average error data from iterations 31 to 40 are shown in Table 8 and Table 9.

Based on the table, it is apparent that the average calculation error after iteration was approximately 1.3 cm, with a maximum error of 3.11 cm. This correction method optimized the propagation path of sound waves by correcting the model simulation data based on the relationship between the main propagation path and the straight-line path. Subsequently, by combining the corrected propagation characteristics with the iterative method, further improvement in the accuracy of localization was achieved.

In the study of partial discharge acoustic signal localization within transformers, various scholars have established models with different sizes and parameters by using different algorithms to achieve localization results with varying degrees of accuracy. The results are shown in Table 10. In comparison, the method adopted in this paper, which combined TDOA and iterative algorithms with model parameters, achieved relatively accurate PD calculation results under comprehensive conditions considering different types and temperatures. The error was also relatively significant compared to many other studies, demonstrating the feasibility of our research method.

Current research widely uses acoustic localization to determine transformer partial discharge locations. Compared to traditional UHF techniques, acoustic methods offer stronger EMI resistance and higher sensitivity, making them valuable for precise calculations. Our study combined models and algorithms, using TDOA as the foundational method. By integrating dynamic distance differences and error iteration algorithms, we achieved more accurate results.

## 6. Conclusions

This paper primarily proposed a novel method for detecting and locating partial discharge signals using a sensor array. The sensors were placed inside the transformer oil tank, resulting in higher localization accuracy. This study modeled the acoustic field of the entire oil-immersed transformer and the built-in sensor array using COMSOL Multiphysics software. The simulation results were obtained through the “electric-force-acoustic” multiphysics coupling method. The paper utilized the time difference of arrival (TDOA) combined with the dynamic distance difference correction method to position the specific location of the PD source. The article concludes with the following conclusions:

1. This study primarily focused on the modeling of the entire transformer, taking into account its complex internal structure as well as different types of defects and operating conditions with the ability to control computational errors within an extremely small range of 1.3 cm.
2. In analyzing the mechanism of partial discharge (PD)-induced acoustic waves, this study combined theoretical analysis with simulation devices. By utilizing the coupling of the “electric-force-acoustic” physical fields, it was confirmed that the acoustic waves generated by PD propagated in the form of sinusoidal oscillations and exhibited exponential decay.
3. Compared to the traditional layout of five-element cross-shaped sensor arrays on the same plane, the z-axis coordinate accuracy in the calculation results was insufficient. Hence, this paper employed a symmetric approach to set a reference value for the z-coordinate, effectively enhancing the overall positioning accuracy.
4. The method proposed in this paper does not account for the influence of the transformer tank on sound wave propagation. Therefore, it is more appropriate for power transformers that are equipped with sensors. Even though they are more costly and difficult to install, this method avoids the computational and attenuation issues associated with transverse waves, thus increasing the localization accuracy.
5. The acoustic wave propagation characteristics under different defects and temperatures were analyzed separately, and this characteristic was validated through specific examples in this study. Utilizing the time difference of arrival (TDOA) combined with dynamic distance difference correction for positioning, the localization results under different defects and temperatures were obtained. Finally, a more accurate localization coordinate was achieved through an error iteration algorithm.

The calculation and positioning accuracy of the novel sensor array in this study had a certain degree of reliability because research of this nature is somewhat limited when using experimental methods and is more suitable for investigation through numerical analysis using FEM. However, acoustic sensors are susceptible to interference from uncertain external sound sources. Consequently, the proposed method for acoustic source localization cannot consider external uncertain interference factors. In the absence of uncertain external sound sources, the presented array and calculation method can provide a theoretical basis for the acoustic localization problem within transformers.

## Figures and Tables

**Table 1:** Transformer geometry model dimensions.

Parameter	Size (m)
Transformer tank (L × W × H)	3 × 2 × 1.5
Inside diameter of high voltage winding	0.183
High voltage winding outside diameter	0.2202
Inside diameter of low voltage winding	0.145
High voltage winding outside diameter	0.173
Core width	1.2848
Core height	1.024
Partial discharge point diameter	0.002

**Table 2:** Properties of the transformer’s internal materials.

Structure	Materials	Density (kg/m^3^)	Sound Velocity (m/s)	Young Modulus (GPa)
Transformer tank	Aluminum	2700	5000	70
Core	Silicon Steel Sheet	7120	5100	198
Coil	Copper	8940	4760	126
Electric insulating oil	/	890	1420	14

**Table 3:** Location coordinates of the actual PD source.

	PD_1_ (m)	PD_2_ (m)	PD_3_ (m)	PD_4_ (m)
α	(−0.25, 0, 0.7)	(−0.5, 0.15, 0.7)	(−0.65, 0.05, 0.8)	(−0.13, 0.15, 0.65)
β	(0.28, −0.1, 0.5)	(−0.1, 0.07, 0.7)	(0.65, −0.05, 0.6)	(0.12, 0.18, 0.80)

**Table 4:** The position coordinates of PD source under different defects are simulated.

Position	Air-Gap Discharge	Surface Discharge	Tip Discharge
PD_1−α_ (m)	(−0.27, −0.09, 0.69)	/	/
PD_2−α_ (m)	(−0.52, 0.14, 0.68)	(−0.46, 0.18, 0.71)	(−0.52, 0.13, 0.68)
PD_3−α_ (m)	(−0.66, 0.07, 0.89)	(−0.69, 0.1, 0.77)	(−0.64, 0.05, 0.77)
PD_4−α_ (m)	(−0.15, 0.08, 0.67)	(−0.17, 0.17, 0.67)	(−0.12, 0.14, 0.657)

**Table 5:** The position coordinates of PD source at different temperatures were simulated.

Temperature	293.15 K	313.15 K	343.15 K	363.15 K
PD_1−β_ (m)	(0.28, −0.14, 0.51)	(0.27, −0.12, 0.52)	(0.28, −0.14, 0.51)	(0.27, −0.13, 0.52)
PD_2−β_ (m)	(−0.07, 0.07, 0.71)	(−0.11, 0.04, 0.69)	(−0.08, 0.08, 0.70)	(−0.09, 0.06, 0.70)
PD_3−β_ (m)	(0.61, −0.04, 0.68)	(0.62, −0.03, 0.69)	(0.71, −0.11, 0.51)	(0.69, −0.05, 0.61)
PD_4−β_ (m)	(0.10, 0.15, 0.80)	(0.07, 0.20, 0.78)	(0.09, 0.18, 0.80)	(0.04, 0.21, 0.77)

**Table 6:** Error between the calculated position and actual position under different defects.

ΔR	Air-Gap Discharge	Surface Discharge	Tip Discharge
PD_1−α_ (m)	0.0927	/	/
PD_2−α_ (m)	0.0300	0.0509	0.0346
PD_3−α_ (m)	0.0927	0.0707	0.0316
PD_4−α_ (m)	0.0754	0.0489	0.0173
Average (m)	0.0727	0.0568	0.0278

**Table 7:** Error between the calculated position and actual position at different temperatures.

ΔR	293.15 K	313.15 K	343.15 K	363.15 K
PD_1−β_ (m)	0.0412	0.0300	0.0412	0.0374
PD_2−β_ (m)	0.0316	0.0331	0.0223	0.0141
PD_3−β_ (m)	0.0900	0.0969	0.1237	0.0412
PD_4−β_ (m)	0.0360	0.0574	0.0300	0.0905
Average (m)	0.0497	0.0544	0.0543	0.0458

**Table 8:** Error between the calculated position and the actual position under different defects after iteration.

ΔR	Air-Gap Discharge	Surface Discharge	Tip Discharge
PD_1−α_ (m)	0.0233	/	/
PD_2−α_ (m)	0.0076	0.0128	0.0087
PD_3−α_ (m)	0.0233	0.0178	0.0079
PD_4−α_ (m)	0.0189	0.0123	0.0044
Average (m)	0.0183	0.0143	0.0070

**Table 9:** Error between the calculated position and the actual position at different temperatures after iteration.

ΔR	293.15 K	313.15 K	343.15 K	363.15 K
PD_1−β_ (m)	0.0104	0.0076	0.0104	0.0094
PD_2−β_ (m)	0.0079	0.0083	0.0056	0.0035
PD_3−β_ (m)	0.0227	0.0243	0.0311	0.0104
PD_4−β_ (m)	0.0090	0.0144	0.0076	0.0228
Average (m)	0.0125	0.0137	0.0137	0.0115

**Table 10:** Comparison of accuracy of computational positioning results in relevant studies.

Model Size (m)	Dimensionality	Location Method	Error	Refs
2.4 × 0.8 × 0.8	3D	TDOA, Levenberg–Marquardt	Less than 5 cm	[31]
3 × 2 × 1.5	3D	TDOA, Dynamic distance difference correction	Less than 3.11 cm	This work
0.32 × 0.32 × 0.32	3D	TDOA, Source-filter model	Less than 1.2 cm	[32]
/	2D	Direct method, Newton–Raphson	Less than 3.26 cm	[33]
5.45 × 2.25 × 2.49	3D	Particle swarm optimization route searching algorithm	Average error of 11.7 cm	[34]

## Data Availability

The data presented in this study are available on request from the corresponding author due to privacy restrictions.
